# Study of the Bias of the Initial Phase Estimation of a Sinewave of Known Frequency in the Presence of Phase Noise

**DOI:** 10.3390/s24123730

**Published:** 2024-06-08

**Authors:** Francisco A. C. Alegria, Lian Xie, Dário Pasadas

**Affiliations:** Instituto de Telecomunicações, Instituto Superior Técnico, Universidade de Lisboa, 1649-004 Lisboa, Portugal; lian.xie@tecnico.ulisboa.pt (L.X.); dpasadas@lx.it.pt (D.P.)

**Keywords:** least squares, sine fitting, initial phase estimation, phase noise, sampling jitter

## Abstract

The estimation of the parameters of a sinusoidal signal is of paramount importance in various applications in the fields of sensors, signal processing, parameter estimation, and device characterization, among others. The presence, in the measurement system, of non-ideal phenomena such as additive noise in the signals, phase noise in the stimulus generation, jitter in the sampling system, frequency error in the experimental setup, among others, leads to increased uncertainty and bias in the estimated quantities obtained by least squares methods and those derived from them. Therefore, from a metrological point of view, it is important to be able to theoretically predict and quantify those uncertainties in order to properly design the measurement system and its parameters, such as the number of samples to acquire or the stimulus signal amplitude to use to minimize the uncertainty in the estimated values. Previous works have shown that the presence of these non-ideal phenomena leads to increased uncertainty and bias in the estimation of the sinewave amplitude. The present work complements this knowledge by focusing specifically on the effect of phase noise and sampling jitter in the bias of the initial phase estimation of a sinusoidal signal of known frequency (three-parameter sine fitting procedure). A theoretical derivation of the bias of initial phase estimation that takes into consideration the presence of phase noise in the sinewave is presented. Since a Taylor series approximation was used where only the first term was retained, it was necessary to validate the analytical derivations with numerical simulations using a Monte Carlo type of procedure. This process was applied to different conditions regarding the phase noise standard deviation, initial phase value, and number of samples. It is concluded that, in most scenarios, initial phase estimation using sine fitting is unbiased in the presence of phase noise or jitter. It is shown, however, that in cases of extremely high phase noise standard deviation and a very low number of samples, a bias occurs.

## 1. Introduction

In almost all areas of engineering, it is valuable to estimate the parameters of various systems or signals [1]. It is often the case that these signals are sinusoidal in time or can be decomposed into a sum of sinewaves. The parameters of a sinewave that may need to be estimated include the amplitude, the offset, the frequency, and the initial phase. The present work focuses on the latter. Estimating the initial phase of an electrical signal is important for measuring various indirect physical quantities, such as displacement [2], strain, acceleration, and power quality [3], among others, as well as in the context of various applications such as sonar, radar [4], and vibration analysis [5]. In addition, phase measurements are essential for characterizing the frequency response of electrical or electronic circuits and instruments. There are also applications in estimating the phase difference between two sinewaves [6] such as electric power calibration or measuring an unknown electrical impedance [7], and estimating the phase difference between voltage and current sinewaves. There are many systems that perform this estimation using specialized hardware circuits [8]. However, there are an increasing number of systems that sample and digitize a signal from the real world and then store and process it digitally on a computer. It is in the latter case that the current work finds its value.

This estimation, carried out on real signals, usually obtained from some kind of sensor, is always affected by the non-ideal phenomena present, which one tries to minimize but cannot completely eliminate. The most recognized one is the presence of additive noise [9], which is often of a thermal origin but can also appear due to other sources, such as interference between systems. As such, this type of noise has a broad frequency spectrum that is, in principle, white, but it often becomes colored due to unintentional or intentional filtering. Since all signals in an electronic system have to be generated somewhere, and this process is also not perfect, phase noise is always present where the phase of the generated sinewave changes randomly due to various reasons, often also related to the presence of thermal noise. It has been shown in the past that the presence of additive noise leads to a bias in the amplitude estimation of a sinewave [10]. This is also the case when jitter is present at the sampling instant [11]. Phase noise in oscillators is also a common non-ideal phenomenon encountered in practice [12]. It is therefore pertinent to ask whether or not these non-ideal phenomena lead to an estimation bias in the case of initial phase estimation. Even a negative result is valuable in the larger context of advancing current scientific knowledge. There is also another effect that contributes to the uncertainty of sinewave parameter estimation, which is the quantization error introduced when the signal of interest is sampled and converted from analogue to digital, which does influence the sine fitting procedure [13].

Elsewhere, the problem of estimating the precision of the sinewave initial phase estimation in the presence of this type of phase noise and jitter [14], as well as other non-ideal phenomena such as additive noise [15], colored noise [16], and frequency error [17], has been addressed. In [15], it was shown that in the case of extremely short record length, there is a bias in the initial phase estimation when additive noise is present.

Here, we focus on the bias of the estimation in the presence of phase noise or sampling jitter. We will conclude that it is, in most situations, null. We will also show that in some very particular cases, it is not. These results were validated using a Monte Carlo type of procedure where the estimation was carried out many times (one million, in this case), with different values of noise, and a confidence value interval was obtained that includes the value 0. It should be noted that this study was conducted assuming that the signal frequency is known. In the future, it will be undoubtedly helpful to also consider a case where the frequency is unknown and must be estimated using different algorithms like, for example, a four-parameter least squares sine fitting algorithm.

## 2. Least Squares Approximation

Before delving into the problem of estimating the initial phase of a sinewave using a least squares procedure, it is important to be clear about what this procedure involves and which mathematical assumptions are made [18,19].

Given a set of data points $\left(t_{i},v_{i}\right)$ for i=1, 2, …, M, where $t_{i}$ is the independent variable value and $v_{i}$ is the observed dependent variable, we want to find a function g(t;a) that depends on a vector of parameters $a=\left(a_{1},a_{2},\;\ldots ,\;a_{n}\right)$ such that the sum of squared residuals is minimized. The residual for each data point is given by the difference between observed value $y_{i}$ and the predicted value $g\left(t_{i};a\right)$.

The goal is to minimize the sum of squared residuals given by

(1) $$S\left(a\right)=\overset{M}{\underset{i=1}{\sum }}{\left[v_{i}-g\left(t_{i};a\right)\right]}^{2},$$

hence the name “least squares”. This is thus an optimization problem where the function to be optimized is S(a), the decision variables are $a=\left(a_{1},a_{2},\;\ldots ,\;a_{n}\right)$, and we look to minimize the value of S(a). In the context of this work, the function *g* is

(2) g(t;A,φ, C)=C+A⋅cos(2πf⋅t+φ),

where the parameter f, the frequency, is assumed to be known. The three parameters of the model that must be determined are the amplitude (*A*), initial phase (φ), and offset (*C*).

It is convenient to rewrite the model as

(3) $$g\left(t;A_{I},A_{Q},\;C\right)=C+A_{I}\cdot \cos\left(2\pi f\cdot t\right)+A_{Q}\cdot \sin\left(2\pi f\cdot t\right),$$

where, instead of the amplitude and initial phase, we use two different parameters called the in-phase amplitude ($A_{I}$) and the in-quadrature amplitude ($A_{Q}$), which are related to the amplitude and initial phase by

(4) $$A_{I}=A\cdot \cos\left(\varphi \right)$$

and

(5) $$A_{Q}=A\cdot \sin\left(\varphi \right).$$

The least squares estimation of the three sinewave parameters are obtained, in matrix form, by

(6) $$\left[\begin{array}{c} {\hat{A}}_{I}\\ {\hat{A}}_{Q}\\ \hat{C} \end{array}\right]={\left(D^{T}D\right)}^{-1}D^{T}\left[\begin{array}{c} v_{1}\\ v_{2}\\ \ldots \\ v_{M} \end{array}\right]$$

where

(7) $$D=\left[\begin{array}{ccc} \cos\left(2\pi f\cdot t_{1}\right) & \sin\left(2\pi f\cdot t_{1}\right) & 1\\ \cos\left(2\pi f\cdot t_{2}\right) & \sin\left(2\pi f\cdot t_{2}\right) & 1\\ \ldots & \ldots & \ldots \\ \cos\left(2\pi f\cdot t_{M}\right) & \sin\left(2\pi f\cdot t_{M}\right) & 1 \end{array}\right].$$

To recover the estimation of the original sinewave parameters, besides the offset $\hat{C}$, we simply use

(8) $$\hat{A}=\sqrt{{\hat{A_{I}}}^{2}+{\hat{A_{Q}}}^{2}}$$

and

(9) $$\hat{\varphi }=\arctan\left(\frac{\hat{A_{Q}}}{\hat{A_{I}}}\right).$$

Note that if ${\hat{A}}_{I}$ turns out to be negative, the constant π is conventionally added to the initial phase estimation. We thus obtain estimates for the sinewave amplitude, initial phase, and offset from the *M* data samples.

The least squares method assumes that the variance of the errors (residuals) is constant across all values of the independent variable (homoscedasticity). If the variance of the errors varies (heteroscedasticity), the method may produce biased estimates. In the present situation, where the independent variable is time, we assume that the variance of the phase noise does not change with time.

Another assumption is that the value of the phase noise at one instant in time is not correlated with the value of the phase noise at a different instant in time, and hence the different residuals are independent of each other.

A third assumption is that the data points cover at least one full period of the sinewave. If this is violated, the initial phase estimation may indeed be biased, as shown in [15].

## 3. Estimating the Initial Phase of a Sinewave

Consider a sinewave with amplitude *A*, initial phase *φ*, offset *C,* and frequency *f*:

(10) x(t)=C+A⋅cos(2πf⋅t+φ).

Furthermore, consider that this signal is sampled at a frequency of $f_{s}$, leading to a set of M samples numbered from i=1 to i=M. The sampled signal values are thus given by

(11) $$x_{i}=C+A\cdot \cos\left(2\pi f\cdot t_{i}+\varphi \right),\;i=1,\ldots ,M$$

where

(12) $$t_{i}=\frac{i-1}{f_{s}},\;i=1,\ldots ,M.$$

In this work, we specifically consider the phenomena of phase noise in the signal generator and the occurrence of sampling jitter in the acquired samples. These two phenomena are considered together since, mathematically, they can be treated simultaneously. The other non-ideal phenomena mentioned have already been considered elsewhere [14].

The sample jitter is represented here with the random variable $\delta_{i}$, such that the sample instants become

(13) $$t_{i}^{\prime }=t_{i}+\delta_{i}.$$

The phase noise, in turn, is represented by the random variable η, such that the value of the samples in the presence of these two types of noise is represented by

(14) $$z_{i}=C+A\cdot \cos\left[2\pi f\cdot \left(t_{i}+\delta_{i}\right)+\varphi +\eta_{i}\right],\;i=1,\ldots ,M.$$

We can then compare this with Equation (10), the ideal sample values. The values of the estimated parameters are obtained using the in-phase amplitude estimative, given by (5), which can be written as (in this case, using $z_{i}$ instead of $v_{i}$ as the dependent variable)

(15) $$\hat{A_{I}}=\frac{2}{M}\overset{M}{\underset{i=1}{\sum }}z_{i}\cdot \cos\left(\omega \cdot t_{i}\right),$$

and the in-quadrature amplitude estimative

(16) $$\hat{A_{Q}}=\frac{2}{M}\overset{M}{\underset{i=1}{\sum }}z_{i}\cdot \sin\left(\omega \cdot t_{i}\right),$$

where ω=2πf. From these, one can obtain the estimated sinewave amplitude given by (7) and (8).

As can easily be seen, any non-ideal phenomena that affects the sample value $z_{i}$ will also affect the amplitude and initial phase estimates, $\hat{A}$ and $\hat{\varphi }$.

There are two random variables in (13), namely $\delta_{i}$ and $\eta_{i}$: jitter in the sampling instant and phase noise in the stimulus signal at the instant of sampling. In most scenarios the phenomena leading to the randomness of these two variables are unrelated. It is therefore justifiable to consider them as independent variables and to describe them mathematically with a single variable, represented here by

(17) $$\theta_{i}=\omega \cdot \delta_{i}+\eta_{i},\;i=1,\ldots ,M.$$

Note that in this work we consider the frequency of the sinusoidal signal, f, to be known. Our model (13) thus becomes

(18) $$z_{i}=C+A\cdot \cos\left(\omega \cdot t_{i}+\varphi +\theta_{i}\right),\;i=1,\ldots ,M.$$

These are the values of the dependent variable to which we want to fit, via least squares, a sinewave model with three unknown parameters.

## 4. Expected Value of the Initial Phase Estimation

Since the estimated initial phase, $\hat{\varphi }$, is a function of the estimated in-phase and in-quadrature amplitudes, we can express the expected value of the initial phase estimation using an approximate expression made of the first term in a Taylor series approximation using Equation (7.20) in [20]:

(19) $$\mu_{\hat{\varphi }}\approx \arctan\left(\frac{\mu_{\hat{A_{Q}}}}{\mu_{\hat{A_{I}}}}\right)+\frac{1}{2}\left(\frac{\partial^{2}\hat{\varphi }}{\partial {\hat{A_{I}}}^{2}}\sigma_{\hat{A_{I}}}^{2}+2\frac{\partial^{2}\hat{\varphi }}{\partial \hat{A_{I}}\partial \hat{A_{Q}}}Cov\left\{\hat{A_{I}},\hat{A_{Q}}\right\}+\frac{\partial^{2}\hat{\varphi }}{\partial {\hat{A_{Q}}}^{2}}\sigma_{\hat{A_{Q}}}^{2}\right),$$

where the partial derivatives should be computed for $\hat{A_{I}}=\mu_{\hat{A_{I}}}$ and $\hat{A_{Q}}=\mu_{\hat{A_{Q}}}$, the expected values of the estimated in-phase and in-quadrature amplitudes, respectively. Next, we must compute the partial derivatives, the variances, and the covariance. In the end, we can bring all these parts together to obtain an expression for the average of the estimated initial phase.

### 4.1. Mean Values of the In-Phase and In-Quadrature Amplitudes

Computing the means of the two estimated amplitudes $\hat{A_{I}}$ and $\hat{A_{Q}}$ is straightforward. From (15), we can deduce

(20) $$\mu_{\hat{A_{Q}}}=\frac{2}{M}\sum_{i=1}^{M}E\left\{z_{i}\right\}\cdot \sin\left(\omega \cdot t_{i}\right).$$

The expected value of the sample values can be obtained from (17). One thus obtains

(21) $$E\left\{z_{i}\right\}=A\cdot E\left\{\cos\left(\omega \cdot t_{i}+\varphi +\theta_{i}\right)\right\}.$$

Assuming that the phase noise is normally distributed, with a mean equal to null, we can write

(22) $$E\left\{\cos\left(a+\xi \right)\right\}=\int_{-\infty }^{\infty }\cos\left(a+\xi \right)\frac{1}{\sqrt{2\pi }\sigma_{\xi }}e^{-\frac{\xi^{2}}{2\sigma_{\xi }^{2}}}d\xi =\cos\left(a\right)e^{-\frac{1}{2}\sigma_{\xi }^{2}},$$

where $a=\omega \cdot t_{i}+\varphi$ and $\xi =\theta_{i}$. This leads to

(23) $$E\left\{z_{i}\right\}=A\cdot \cos\left(\omega \cdot t_{i}+\varphi \;\right)e^{-\frac{1}{2}\sigma_{\theta }^{2}}.$$

Inserting this into (19) leads to

(24) $$\mu_{\hat{A_{Q}}}=\frac{2}{M}\sum_{i=1}^{M}A\cdot E\left\{\cos\left(\omega \cdot t_{i}+\varphi \;\right)e^{-\frac{1}{2}\sigma_{\theta }^{2}}\right\}\cdot \sin\left(\omega \cdot t_{i}\right).$$

Making use of

(25) $$\cos\left(a\right)\sin\left(b\right)=\frac{1}{2}\sin\left(a+b\right)+\frac{1}{2}\sin\left(a-b\right)$$

leads to

(26) $$\mu_{\hat{A_{Q}}}=\frac{A}{M}e^{-\frac{1}{2}\sigma_{\theta }^{2}}\sum_{i=1}^{M}\sin\left(2\omega \cdot t_{i}+\varphi \right)+\frac{A}{M}e^{-\frac{1}{2}\sigma_{\theta }^{2}}\sum_{i=1}^{M}\sin\left(\varphi \right).$$

Since the first summation covers an integer number of periods, it results in a null value. The second summation results in M⋅sin(φ). Hence, we obtain

(27) $$\mu_{\hat{A_{Q}}}=A\cdot \sin\left(\varphi \right)\cdot e^{-\frac{1}{2}\sigma_{\theta }^{2}}.$$

Repeating the same computations for the in-phase amplitude leads to

(28) $$\mu_{\hat{A_{I}}}=A\cdot \cos\left(\varphi \right)\cdot e^{-\frac{1}{2}\sigma_{\theta }^{2}}.$$

We now move on to the computation of the second raw moments using a similar procedure.

### 4.2. Second Raw Moment of the In-Phase and In-Quadrature Amplitudes

Here, we determine the second raw moment of the in-phase amplitude, which will later enable us to compute the variance of those amplitudes. We obtain, from (14),

(29) $$E\;\left\{{\hat{A_{I}}}^{2}\right\}=E\left\{{\left[\frac{2}{M}\sum_{i=1}^{M}z_{i}\cos\left(\omega \cdot t_{i}\right)\right]}^{2}\right\}.$$

By computing the square of square bracket, we obtain

(30) $$E\;\left\{{\hat{A_{I}}}^{2}\right\}=\frac{4}{M^{2}}E\left\{\sum_{i,j}\begin{array}{c} z_{i}z_{j}cos\left(\omega \cdot t_{i}\right)cos\left(\omega \cdot t_{j}\right) \end{array}\right\}.$$

The expected value can be moved inside the double summation, leading to

(31) $$E\;\left\{{\hat{A_{I}}}^{2}\right\}=\frac{4}{M^{2}}\sum_{i,j}E\left\{z_{i}z_{j}\right\}\cos\left(\omega \cdot t_{i}\right)\cos\left(\omega \cdot t_{j}\right).$$

To compute $E\left\{z_{i}z_{j}\right\}$, we start with the expression of $z_{i}$ as given in (17) and obtain

(32) $$E\left\{z_{i}z_{j}\right\}=E\left\{\left[A\cos\left(\omega \cdot t_{i}+\varphi +\theta_{i}\right)\right]\cdot \left[A\cos\left(\omega \cdot t_{j}+\varphi +\theta_{j}\right)\right]\right\}.$$

Multiplying the terms in the two square brackets leads to

(33) $$E\left\{z_{i}z_{j}\right\}=E\left\{A^{2}\cos\left(\omega \cdot t_{i}+\varphi +\theta_{i}\right)\cos\left(\omega \cdot t_{j}+\varphi +\theta_{j}\right)\right\}.$$

By making use of

(34) $$\cos\left(a\right)\cos\left(b\right)=\frac{1}{2}\cos\left(a+b\right)+\frac{1}{2}\cos\left(a-b\right),$$

we can write

(35) $$E\left\{z_{i}z_{j}\right\}=E\left\{\begin{array}{c} \frac{A^{2}}{2}\cos\left(\omega \cdot t_{i}+\omega \cdot t_{j}+2\varphi +\theta_{i}+\theta_{j}\right)+\\ \frac{A^{2}}{2}\cos\left(\omega \cdot t_{i}-\omega \cdot t_{j}+\theta -\theta_{j}\right) \end{array}\right\}.$$

Using the fact that the expected value of the sum is equal to the sum of expected values and that the expected value of a constant times a random variable is equal to that same constant times the expected value of the random variable, we can deduce that

(36) $$E\left\{z_{i}z_{j}\right\}=\frac{A^{2}}{2}E\left\{\cos\left(\omega \cdot t_{i}+\omega \cdot t_{j}+2\varphi +\eta_{i}+\eta_{j}\right)\right\}+\;\frac{A^{2}}{2}E\left\{\cos\left(\omega \cdot t_{i}-\omega \cdot t_{j}+\eta_{i}-\eta_{j}\right)\right\}.$$

Writing two different expressions for the cases where the indexes are different (called $a_{ij}$) and are the same (called $b_{i}$) leads to

(37) $$\begin{array}{ll} a_{ij}=E{\left\{z_{i}z_{j}\right\}}_{i\neq j} & =\frac{A^{2}}{2}E\left\{\cos\left(\omega \cdot t_{i}+\omega \cdot t_{j}+2\varphi +\theta_{i}+\theta_{j}\right)\right\}\\ & +\frac{A^{2}}{2}E\left\{\cos\left(\omega \cdot t_{i}-\omega \cdot t_{j}+\theta_{i}-\theta_{j}\right)\right\} \end{array}$$

and

(38) $$b_{i}=E{\left\{z_{i}z_{j}\right\}}_{i=j}=\frac{A^{2}}{2}E\left\{\cos\left(2\omega \cdot t_{i}+2\varphi +2\theta_{i}\right)\right\}+\frac{A^{2}}{2}.$$

We are now able to compute the expected values when considering that $\theta_{i}$ and $\theta_{j}$ are two independent random variables (if i≠j), which are normally distributed with null mean and the same variance $\sigma_{\theta }^{2}$. We are going to make use of the definition of the expected value,

(39) $$E\left\{\cos\left(k+\xi \right)\right\}=\int_{-\infty }^{\infty }\cos\left(k+\xi \right)\frac{1}{\sqrt{2\pi }\sigma_{\xi }}e^{-\frac{\xi^{2}}{2\sigma_{\xi }^{2}}}d\xi =\cos\left(k\right)e^{-\frac{1}{2}\sigma_{\xi }^{2}},$$

where k is a generic constant and ξ is a random variable. The coefficient $a_{ij}$, given in (35), when considering that $\xi =\theta_{i}\pm \theta_{j}$ and thus that $\sigma_{\xi }^{2}=2\sigma_{\theta }^{2}$, can be written as

(40) $$a_{ij}=\frac{A^{2}}{2}\cos\left(\omega \cdot t_{i}+\omega \cdot t_{j}+2\varphi \right)e^{-\sigma_{\theta }^{2}}+\frac{A^{2}}{2}\cos\left(\omega \cdot t_{i}-\omega \cdot t_{j}\right)e^{-\sigma_{\theta }^{2}}.$$

Equation (36), when considering that $\xi =2\theta_{i}$ and thus that $\sigma_{\xi }^{2}=4\sigma_{\theta }^{2}$, becomes

(41) $$b_{i}=\frac{A^{2}}{2}+\frac{A^{2}}{2}\cos\left(2\omega \cdot t_{i}+2\varphi \right)e^{-2\sigma_{\theta }^{2}}.$$

The analytical expression for the expected value $E\left\{z_{i}z_{j}\right\}$ is different depending on whether the two indexes are the same ($a_{ij}$ ) or not ($b_{i}$).

(42) $$E\left\{{\hat{A_{I}}}^{2}\right\}=\frac{4}{M^{2}}\sum_{i,j}\left[\{\begin{array}{c} a_{ij},\;\;i\neq j\\ b_{i},\;\;\;i=j \end{array}\right]\cdot \cos\left(\omega t_{i}\right)\cos\left(\omega t_{j}\right).$$

This can be written as an addition of two summations:

(43) $$E\left\{{\hat{A_{I}}}^{2}\right\}=\frac{4}{M^{2}}\sum_{i\neq j}a_{ij}\cos\left(\omega \cdot t_{i}\right)\cos\left(\omega \cdot t_{j}\right)+\frac{4}{M^{2}}\sum_{i}b_{i}\cos^{2}\left(\omega \cdot t_{i}\right).$$

To compute the first summation, the one involving $a_{ij}$, it is convenient to have a complete summation, that is, one where the term i=j is not missing. To achieve this, we use the complete summation, as desired, and subtract, using a single summation, the terms that should not be in the double summation. We can thus write the expected value of the square of the in-phase amplitude as

(44) $$E\left\{{\hat{A_{I}}}^{2}\right\}=\frac{4}{M^{2}}\sum_{i,j}a_{ij}\cos\left(\omega \cdot t_{i}\right)\cos\left(\omega \cdot t_{j}\right)-\frac{4}{M^{2}}\sum_{i}a_{ii}\cos^{2}\left(\omega \cdot t_{i}\right)+\;\frac{4}{M^{2}}\sum_{i}b_{i}\cos^{2}\left(\omega \cdot t_{i}\right).$$

The same procedure was used in [14]. Applying (32) in the first term of (42) leads to

(45) $$E\left\{{\hat{A_{I}}}^{2}\right\}=\frac{2}{M^{2}}\underset{S_{a}}{\underset{︸}{\sum_{i,j}a_{ij}\cos\left(\omega \cdot t_{i}+\omega \cdot t_{j}\right)}}+\frac{2}{M^{2}}\underset{S_{b}}{\underset{︸}{\sum_{i,j}a_{ij}\cos\left(\omega \cdot t_{i}-\omega \cdot t_{j}\right)}}-\;\frac{4}{M^{2}}\underset{S_{c}}{\underset{︸}{\sum_{i}a_{ii}\cos^{2}\left(\omega \cdot t_{i}\right)}}+\frac{4}{M^{2}}\underset{S_{d}}{\underset{︸}{\sum_{i}b_{i}\cos^{2}\left(\omega \cdot t_{i}\right)}}.$$

Note that each term has been named $S_{a}$, $S_{b}$, $S_{c}$, and $S_{d}$, such that

(46) $$E\left\{{\hat{A_{I}}}^{2}\right\}=\frac{2}{M^{2}}s_{a}+\frac{2}{M^{2}}s_{b}-\frac{4}{M^{2}}s_{c}+\frac{4}{M^{2}}s_{d}.$$

Inserting the coefficients $a_{ij}$ and $b_{i}$ into each of the summation terms, starting with $s_{a}$, produces

(47) $$s_{a}=\sum_{i,j}a_{ij}\cos\left(\omega \cdot t_{i}+\omega t_{j}\right),$$

and using $a_{ij}$, given by (38), leads to

(48) $$s_{a}=\sum_{i,j}\frac{A^{2}}{2}\cos\left(\omega \cdot t_{i}+\omega \cdot t_{j}+2\varphi \right)e^{-\sigma_{\theta }^{2}}\cos\left(\omega \cdot t_{i}+\omega \cdot t_{j}\right)+\;\sum_{i,j}\frac{A^{2}}{2}\cos\left(\omega \cdot t_{i}-\omega \cdot t_{j}\right)e^{-\sigma_{\theta }^{2}}\cos\left(\omega \cdot t_{i}+\omega \cdot t_{j}\right).$$

Carrying out the product of the cosine functions using (32) leads to

(49) $$s_{a}=\sum_{i,j}\frac{A^{2}}{4}\cos\left(2\omega \cdot t_{i}+\omega \cdot t_{j}+2\varphi \right)e^{-\sigma_{\theta }^{2}}+\sum_{i,j}\frac{A^{2}}{4}\cos\left(2\varphi \right)e^{-\sigma_{\theta }^{2}}+\;\sum_{i,j}\frac{A^{2}}{4}\cos\left(2\omega \cdot t_{i}\right)e^{-\sigma_{\theta }^{2}}+\sum_{i,j}\frac{A^{2}}{4}\cos\left(-2\omega \cdot t_{j}\right)e^{-\sigma_{\theta }^{2}}.$$

Considering that the summations of the first, third, and fourth terms occur over an integer number of periods, they result in a null value. Only the second term remains:

(50) $$s_{a}=\frac{A^{2}M^{2}}{4}\cos\left(2\varphi \right)e^{-\sigma_{\theta }^{2}}.$$

Moving now to $s_{b}$, given in (43), we can deduce that

(51) $$s_{b}=\sum_{i,j}a_{ij}\cos\left(\omega \cdot t_{i}-\omega \cdot t_{j}\right),$$

and making use of $a_{ij}$, given in (38), leads to

(52) $$s_{b}=\sum_{i,j}\frac{A^{2}}{2}\cos\left(\omega \cdot t_{i}+\omega \cdot t_{j}+2\varphi \right)e^{-\sigma_{\theta }^{2}}\cos\left(\omega \cdot t_{i}-\omega \cdot t_{j}\right)+\;\sum_{i,j}\frac{A^{2}}{2}\cos\left(\omega \cdot t_{i}-\omega \cdot t_{j}\right)e^{-\sigma_{\theta }^{2}}\cos\left(\omega \cdot t_{i}-\omega \cdot t_{j}\right).$$

Multiplying the cosines using (32) leads to

(53) $$s_{b}=\sum_{i,j}\frac{A^{2}}{4}\cos\left(2\omega \cdot t_{i}+2\varphi \right)e^{-\sigma_{\theta }^{2}}+\sum_{i,j}\frac{A^{2}}{4}\cos\left(2\omega \cdot t_{j}+2\varphi \right)e^{-\sigma_{\theta }^{2}}+\;\sum_{i,j}\frac{A^{2}}{4}\cos\left(2\omega \cdot t_{i}-2\omega \cdot t_{j}\right)e^{-\sigma_{\theta }^{2}}+\sum_{i,j}\frac{A^{2}}{4}e^{-\sigma_{\theta }^{2}}.$$

Since the first three summations occur over an integer number of periods of a sinusoid, they result in 0. The fourth one does not. By using the fact that the double summation of a constant is just that constant multiplied by the number of terms of the summation, $M^{2}$, we obtain

(54) $$s_{b}=\frac{A^{2}M^{2}}{4}e^{-\sigma_{\theta }^{2}}.$$

We can now tackle $s_{c}$, as defined in (43):

(55) $$s_{c}=\sum_{i}a_{ii}\cos^{2}\left(\omega \cdot t_{i}\right),$$

By inserting $a_{ii}$, given in (38), when i=j, we obtain

(56) $$a_{ii}=\frac{A^{2}}{2}\cos\left(2\omega \cdot t_{i}+2\varphi \right)e^{-\sigma_{\theta }^{2}}+\frac{A^{2}}{2}e^{-\sigma_{\theta }^{2}},$$

This leads to

(57) $$s_{c}=\sum_{i}\frac{A^{2}}{2}\cos\left(2\omega \cdot t_{i}+2\varphi \right)e^{-\sigma_{\theta }^{2}}\cos^{2}\left(\omega \cdot t_{i}\right)+\sum_{i}\frac{A^{2}}{2}e^{-\sigma_{\theta }^{2}}\cos^{2}\left(\omega \cdot t_{i}\right).$$

Using

(58) $$\cos^{2}\left(x\right)=\frac{1}{2}+\frac{1}{2}\cos\left(2x\right)$$

results in

(59) $$s_{c}=\sum_{i}\frac{A^{2}}{4}\cos\left(2\omega \cdot t_{i}+2\varphi \right)e^{-\sigma_{\theta }^{2}}+\sum_{i}\frac{A^{2}}{4}\cos\left(2\omega \cdot t_{i}+2\varphi \right)e^{-\sigma_{\theta }^{2}}\cos\left(2\omega \cdot t_{i}\right)+\;\sum_{i}\frac{A^{2}}{2}e^{-\sigma_{\theta }^{2}}\cos^{2}\left(\omega \cdot t_{i}\right).$$

Using (32) in the second term leads to

(60) $$s_{c}=\sum_{i}\frac{A^{2}}{4}\cos\left(2\omega \cdot t_{i}+2\varphi \right)e^{-\sigma_{\theta }^{2}}+\sum_{i}\frac{A^{2}}{8}\cos\left(4\omega \cdot t_{i}+2\varphi \right)e^{-\sigma_{\theta }^{2}}+\;\sum_{i}\frac{A^{2}}{8}\cos\left(2\varphi \right)e^{-\sigma_{\theta }^{2}}+\sum_{i}\frac{A^{2}}{2}e^{-\sigma_{\theta }^{2}}\cos^{2}\left(\omega \cdot t_{i}\right).$$

The first two summations result in a null value. Notice that, in the third one, the argument does not depend on i. We can thus multiply its contents by the number of terms, which is M, leading to

(61) $$s_{c}=\frac{A^{2}M}{8}\cos\left(2\varphi \right)e^{-\sigma_{\theta }^{2}}+\sum_{i}\frac{A^{2}}{2}e^{-\sigma_{\theta }^{2}}\cos^{2}\left(\omega \cdot t_{i}\right).$$

Using (56) in the remaining summation results in

(62) $$s_{c}=\frac{A^{2}M}{8}\cos\left(2\varphi \right)e^{-\sigma_{\theta }^{2}}+\sum_{i}\frac{A^{2}}{4}e^{-\sigma_{\theta }^{2}}+\sum_{i}\frac{A^{2}}{4}e^{-\sigma_{\theta }^{2}}\cos\left(2\omega \cdot t_{i}\right).$$

The contents of the first summation are independent of i, and the second summation is 0. We thus obtain

(63) $$s_{c}=\frac{A^{2}M}{8}\cos\left(2\varphi \right)e^{-\sigma_{\theta }^{2}}+\frac{A^{2}M}{4}e^{-\sigma_{\theta }^{2}}.$$

The final term, $s_{d}$, from expression (43), can be written as

(64) $$s_{d}=\sum_{i}b_{i}\cos^{2}\left(\omega \cdot t_{i}\right).$$

Inserting $b_{i}$, given by (39), results in

(65) $$s_{d}=\sum_{i}\frac{A^{2}}{2}\cos^{2}\left(\omega \cdot t_{i}\right)+\sum_{i}\frac{A^{2}}{2}\cos\left(2\omega \cdot t_{i}+2\varphi \right)e^{-2\sigma_{\theta }^{2}}\cos^{2}\left(\omega \cdot t_{i}\right).$$

Using (56) leads to

(66) $$s_{d}=\sum_{i}\frac{A^{2}}{4}+\sum_{i}\frac{A^{2}}{4}\cos\left(2\omega \cdot t_{i}\right)+\sum_{i}\frac{A^{2}}{4}\cos\left(2\omega \cdot t_{i}+2\varphi \right)e^{-2\sigma_{\theta }^{2}}+\;\sum_{i}\frac{A^{2}}{4}\cos\left(2\omega \cdot t_{i}+2\varphi \right)e^{-2\sigma_{\theta }^{2}}\cos\left(\omega \cdot t_{i}\right).$$

Using (32) in the last term leads to

(67) $$s_{d}=\sum_{i}\frac{A^{2}}{4}+\sum_{i}\frac{A^{2}}{4}\cos\left(2\omega \cdot t_{i}\right)+\sum_{i}\frac{A^{2}}{4}\cos\left(2\omega \cdot t_{i}+2\varphi \right)e^{-2\sigma_{\theta }^{2}}+\;\sum_{i}\frac{A^{2}}{8}\cos\left(4\omega \cdot t_{i}+2\varphi \right)e^{-2\sigma_{\theta }^{2}}+\sum_{i}\frac{A^{2}}{8}\cos\left(2\varphi \right)e^{-2\sigma_{\theta }^{2}},$$

which can be simplified by again using the fact that the summation over an integer number of periods is 0, leading to

(68) $$s_{d}=\frac{A^{2}M}{4}+\frac{A^{2}M}{8}\cos\left(2\varphi \right)e^{-2\sigma_{\theta }^{2}}.$$

Inserting the four terms just derived, $s_{a}$, $s_{b}$, $s_{c}$, and $s_{d}$, back into (43) leads to

(69) $$E\left\{{\hat{A_{I}}}^{2}\right\}=\frac{2}{M^{2}}\left[\frac{A^{2}M^{2}}{4}\cos\left(2\varphi \right)e^{-\sigma_{\theta }^{2}}\right]+\frac{2}{M^{2}}\left[\frac{A^{2}M^{2}}{4}e^{-\sigma_{\theta }^{2}}\right]-\frac{4}{M^{2}}\left[\frac{A^{2}M}{8}\cos\left(2\varphi \right)e^{-\sigma_{\theta }^{2}}+\frac{A^{2}M}{4}e^{-\sigma_{\theta }^{2}}\right]+\;\frac{4}{M^{2}}\left[\frac{A^{2}M}{4}+\frac{A^{2}M}{8}\cos\left(2\varphi \right)e^{-2\sigma_{\theta }^{2}}\right].$$

By simplifying, we obtain

(70) $$E\left\{{\hat{A_{I}}}^{2}\right\}=\frac{A^{2}}{M}+\frac{A^{2}}{2}\left[\cos\left(2\varphi \right)+1-\frac{1}{M}\cos\left(2\varphi \right)-\frac{2}{M}\right]e^{-\sigma_{\theta }^{2}}+\frac{A^{2}}{2M}\cos\left(2\varphi \right)e^{-2\sigma_{\theta }^{2}}.$$

Repeating the same procedure for the in-quadrature amplitude results in

(71) $$E\left\{{\hat{A_{Q}}}^{2}\right\}=\frac{A^{2}}{M}+\frac{A^{2}}{2}\left[-\cos\left(2\varphi \right)+1+\frac{1}{M}\cos\left(2\varphi \right)-\frac{2}{M}\right]e^{-\sigma_{\theta }^{2}}-\frac{A^{2}}{2M}\cos\left(2\varphi \right)e^{-2\sigma_{\theta }^{2}},$$

which differs from the expected value of the in-phase amplitude due to the minus signs multiplying the cosine functions.

Having computed the expected values and the second raw moments, we can move on to the computation of the variances.

### 4.3. Variances of the In-Phase and In-Quadrature Amplitudes

In order to compute the variance of the in-phase and in-quadrature amplitudes we subtract from second raw moment in (68), the expected value given in (26), leading to

(72) $$\sigma_{\hat{A_{I}}}^{2}=\frac{A^{2}}{M}+\frac{A^{2}}{2}\left[\cos\left(2\varphi \right)+1-\frac{1}{M}\cos\left(2\varphi \right)-\frac{2}{M}\right]e^{-\sigma_{\theta }^{2}}+\frac{A^{2}}{2M}\cos\left(2\varphi \right)e^{-2\sigma_{\theta }^{2}}-\;{\left[A\cos\left(\varphi \right)e^{-\frac{1}{2}\sigma_{\theta }^{2}}\right]}^{2}.$$

Applying (56) to the last term results in

(73) $$\sigma_{\hat{A_{I}}}^{2}=\frac{A^{2}}{M}+\frac{A^{2}}{2}\left[\cos\left(2\varphi \right)+1-\frac{1}{M}\cos\left(2\varphi \right)-\frac{2}{M}\right]e^{-\sigma_{\theta }^{2}}+\frac{A^{2}}{2M}\cos\left(2\varphi \right)e^{-2\sigma_{\theta }^{2}}-\;\frac{A^{2}}{2}\left[\cos\left(2\varphi \right)+1\right]e^{-\sigma_{\theta }^{2}}.$$

Carrying out some simplifications leads to

(74) $$\sigma_{\hat{A_{I}}}^{2}=\frac{A^{2}}{M}-\frac{A^{2}}{2M}\left[2+\cos\left(2\varphi \right)\right]e^{-\sigma_{\theta }^{2}}+\frac{A^{2}}{2M}\cos\left(2\varphi \right)e^{-2\sigma_{\theta }^{2}}.$$

In the case of the in-quadrature amplitude, given by (15), we can see that it has a sine function in place of the cosine function that one can observe in (14). Doing a similar derivation will lead to the following:

(75) $$\sigma_{\hat{A_{Q}}}^{2}=\frac{A^{2}}{M}-\frac{A^{2}}{2M}\left[2-\cos\left(2\varphi \right)\right]e^{-\sigma_{\theta }^{2}}-\frac{A^{2}}{2M}\cos\left(2\varphi \right)e^{-2\sigma_{\theta }^{2}},$$

which is very similar to the variance obtained previously for the in-phase amplitude, given in (72), but with a minus sign before the two cos(2φ) terms. We can thus write them in a different form using

(76) $$\sigma_{\hat{A_{I}}}^{2}=P+R$$

and

(77) $$\sigma_{\hat{A_{Q}}}^{2}=P-R,$$

where

(78) $$P=\frac{A^{2}}{M}\left(1-e^{-\sigma_{\theta }^{2}}\right)$$

and

(79) $$R=-\frac{A^{2}}{2M}\cos\left(2\varphi \right)e^{-\sigma_{\theta }^{2}}+\frac{A^{2}}{2M}\cos\left(2\varphi \right)e^{-2\sigma_{\theta }^{2}},$$

which is equivalent to

(80) $$R=\frac{A^{2}}{2M}\cos\left(2\varphi \right)\left(e^{-2\sigma_{\theta }^{2}}-e^{-\sigma_{\theta }^{2}}\right).$$

We have thus, at this point, determined the variances of the in-phase and in-quadrature amplitudes of the sinewave as a function of the sinusoid’s amplitude (A), initial phase (φ), number of acquired samples (M), and amount of phase noise standard deviation ($\sigma_{\theta }$):

(81) $$\sigma_{\hat{A_{I}}}^{2}=\frac{A^{2}}{M}\left(1-e^{-\sigma_{\theta }^{2}}\right)+\frac{A^{2}}{2M}\cos\left(2\varphi \right)\left(e^{-2\sigma_{\theta }^{2}}-e^{-\sigma_{\theta }^{2}}\right)$$

and

(82) $$\sigma_{\hat{A_{Q}}}^{2}=\frac{A^{2}}{M}\left(1-e^{-\sigma_{\theta }^{2}}\right)-\frac{A^{2}}{2M}\cos\left(2\varphi \right)\left(e^{-2\sigma_{\theta }^{2}}-e^{-\sigma_{\theta }^{2}}\right).$$

It is time to determine the covariance of the two amplitude components.

### 4.4. Co-Variance of the In-Phase and In-Quadrature Amplitudes

To determine the covariance between $\hat{A_{I}}$ and $\hat{A_{Q}}$, we use

(83) $$Cov\left\{\hat{A_{I}},\hat{A_{Q}}\right\}=E\left\{\hat{A_{I}}\hat{\cdot A_{Q}}\right\}-E\left\{\hat{A_{I}}\right\}\cdot E\left\{\hat{A_{Q}}\right\}.$$

The expected value of the product of the two estimated amplitudes is

(84) $$E\left\{\hat{A_{I}}\hat{\cdot A_{Q}}\right\}=\frac{4}{M^{2}}E\left\{\sum_{i,j}\begin{array}{c} z_{i}z_{j}cos\left(\omega_{a}t_{i}\right)\sin\left(\omega_{a}t_{j}\right) \end{array}\right\}.$$

Repeating what the process used earlier for $E\left\{{\hat{A_{I}}}^{2}\right\}$, given by (28), but with a sine function in place of the second cosine function leads to

(85) $$E\left\{\hat{A_{I}}\hat{\cdot A_{Q}}\right\}=\frac{A^{2}}{2}\sin\left(2\varphi \right)\left(1-\frac{1}{M}\right)e^{-\sigma_{\theta }^{2}}+\frac{A^{2}}{2M}\sin\left(2\varphi \right)e^{-2\sigma_{\theta }^{2}}.$$

Inserting this into expression (81), together with (25) and (26), leads to the following:

(86) $$Cov\left\{\hat{A_{I}},\hat{A_{Q}}\right\}=\frac{A^{2}}{2}\sin\left(2\varphi \right)\left(1-\frac{1}{M}\right)e^{-\sigma_{\theta }^{2}}+\frac{A^{2}}{2M}\sin\left(2\varphi \right)e^{-2\sigma_{\theta }^{2}}-\;\left[A\cdot \cos\left(\varphi \right)e^{-\frac{1}{2}\sigma_{\theta }^{2}}\right]\cdot \left[A\cdot \sin\left(\varphi \right)e^{-\frac{1}{2}\sigma_{\theta }^{2}}\right].$$

Carrying out some simplifications leads to

(87) $$Cov\left\{\hat{A_{I}},\hat{A_{Q}}\right\}=\frac{A^{2}}{2M}\sin\left(2\varphi \right)\left(e^{-\sigma_{\theta }^{2}}-e^{-2\sigma_{\theta }^{2}}\right).$$

Notice the similarity between this expression and the one defined earlier for R, in (78).

### 4.5. Partial Derivatives

Computing the second order partial derivatives of $\hat{\varphi }$, given by (8), leads to

(88) $$\frac{\partial^{2}\hat{\varphi }}{\partial {\hat{A_{I}}}^{2}}=2\frac{\frac{\mu_{\hat{A_{Q}}}}{\mu_{\hat{A_{I}}}}}{\mu_{\hat{A_{I}}}^{2}+\mu_{\hat{A_{Q}}}^{2}}-2\frac{\frac{\mu_{\hat{A_{Q}}}^{3}}{\mu_{\hat{A_{I}}}}}{{\left(\mu_{\hat{A_{I}}}^{2}+\mu_{\hat{A_{Q}}}^{2}\right)}^{2}},$$

and

(89) $$\frac{\partial^{2}\hat{\varphi }}{\partial {\hat{A_{Q}}}^{2}}=-2\frac{\mu_{\hat{A_{I}}}\cdot \mu_{\hat{A_{Q}}}}{{\left(\mu_{\hat{A_{I}}}^{2}+\mu_{\hat{A_{Q}}}^{2}\right)}^{2}}.$$

The second-order derivatives of the estimated initial phase relative to the in-phase amplitude, given by (86), becomes, after inserting (25) and (26),

(90) $$\frac{\partial^{2}\hat{\varphi }}{\partial {\hat{A_{I}}}^{2}}=\frac{2}{A^{2}}\cdot \frac{1}{e^{-\sigma_{\theta }^{2}}}\cdot \left[\frac{\frac{\sin\left(\varphi \right)}{\cos\left(\varphi \right)}}{\cos^{2}\left(\varphi \right)+\sin^{2}\left(\varphi \right)}-\frac{\frac{\sin^{3}\left(\varphi \right)}{\cos\left(\varphi \right)}}{{\left(\cos^{2}\left(\varphi \right)+\sin^{2}\left(\varphi \right)\right)}^{2}}\right],$$

which simplifies to

(91) $$\frac{\partial^{2}\hat{\varphi }}{\partial {\hat{A_{I}}}^{2}}=\frac{2}{A^{2}}\cdot \frac{1}{e^{-\sigma_{\theta }^{2}}}\cdot \frac{\sin\left(\varphi \right)\left[1-\sin^{2}\left(\varphi \right)\right]}{\cos\left(\varphi \right)},$$

which further simplifies to

(92) $$\frac{\partial^{2}\hat{\varphi }}{\partial {\hat{A_{I}}}^{2}}=\frac{1}{A^{2}}\cdot e^{\sigma_{\theta }^{2}}\cdot \sin\left(2\varphi \right).$$

Focusing now on the second-order derivative of the in-quadrature component, given by (87), leads, after inserting (25) and (26) and simplifying, to

(93) $$\frac{\partial^{2}\hat{\varphi }}{\partial {\hat{A_{Q}}}^{2}}=-\frac{1}{A^{2}}\cdot e^{\sigma_{\theta }^{2}}\cdot \sin\left(2\varphi \right).$$

Note that this has a symmetrical relationship with (90).

Next, the third second-order derivative present in (18) is, as shown in (8),

(94) $$\frac{\partial^{2}\hat{\varphi }}{\partial \hat{A_{I}}\partial \hat{A_{Q}}}=2\frac{\mu_{\hat{A_{Q}}}^{2}}{{\left(\mu_{\hat{A_{I}}}^{2}+\mu_{\hat{A_{Q}}}^{2}\right)}^{2}}-\frac{1}{\mu_{\hat{A_{I}}}^{2}+\mu_{\hat{A_{Q}}}^{2}}.$$

Inserting (25) and (26) leads to

(95) $$\frac{\partial^{2}\hat{\varphi }}{\partial \hat{A_{I}}\partial \hat{A_{Q}}}=2\frac{A^{2}\cdot \sin^{2}\left(\varphi \right)\cdot e^{-\sigma_{\theta }^{2}}}{{\left(A^{2}\cdot \cos^{2}\left(\varphi \right)\cdot e^{-\sigma_{\theta }^{2}}+A^{2}\cdot \sin^{2}\left(\varphi \right)\cdot e^{-\sigma_{\theta }^{2}}\right)}^{2}}-\frac{1}{A^{2}\cdot \cos^{2}\left(\varphi \right)\cdot e^{-\sigma_{\theta }^{2}}+A^{2}\cdot \sin^{2}\left(\varphi \right)\cdot e^{-\sigma_{\theta }^{2}}}.$$

After some simplification, we obtain

(96) $$\frac{\partial^{2}\hat{\varphi }}{\partial \hat{A_{I}}\partial \hat{A_{Q}}}=\frac{2}{A^{2}}\sin^{2}\left(\varphi \right)\cdot e^{\sigma_{\theta }^{2}}-\frac{1}{A^{2}}e^{\sigma_{\theta }^{2}}.$$

Finaly, using the trigonometric relation $2\sin^{2}\left(\varphi \right)=1-\cos\left(2\varphi \right)$, we obtain

(97) $$\frac{\partial^{2}\hat{\varphi }}{\partial \hat{A_{I}}\partial \hat{A_{Q}}}=-\frac{1}{A^{2}}\cdot e^{\sigma_{\theta }^{2}}\cdot \cos\left(2\varphi \right)$$

which concludes the determination of the partial derivatives.

### 4.6. Bringing It All Together

At this point, we are able to bring together all the terms in (18) and obtain results regarding the bias of the estimated initial phase of a sinewave in the presence of phase noise and/or jitter. Inserting the expected values of the in-phase and in-quadrature amplitudes, given in (25) and (26), leads to

(98) $$\mu_{\hat{\varphi }}\approx \varphi +\frac{1}{2}\left(\frac{\partial^{2}\hat{\varphi }}{\partial {\hat{A_{I}}}^{2}}\sigma_{\hat{A_{I}}}^{2}+2\frac{\partial^{2}\hat{\varphi }}{\partial \hat{A_{I}}\partial \hat{A_{Q}}}Cov\left\{\hat{A_{I}},\hat{A_{Q}}\right\}+\frac{\partial^{2}\hat{\varphi }}{\partial {\hat{A_{Q}}}^{2}}\sigma_{\hat{A_{Q}}}^{2}\right).$$

Inserting the second-order derivatives given in (90), (91), and (95) leads to

(99) $$\mu_{\hat{\varphi }}\approx \varphi +\frac{1}{2}\left\{\begin{array}{c} \left[\frac{1}{A^{2}}\cdot e^{\sigma_{\theta }^{2}}\cdot \sin\left(2\varphi \right)\right]\cdot \sigma_{\hat{A_{I}}}^{2}+\\ 2\cdot \left[\frac{-1}{A^{2}}\cdot e^{\sigma_{\theta }^{2}}\cdot \cos\left(2\varphi \right)\right]\cdot Cov\left\{\hat{A_{I}},\hat{A_{Q}}\right\}+\\ \left[-\frac{1}{A^{2}}\cdot e^{\sigma_{\theta }^{2}}\cdot \sin\left(2\varphi \right)\right]\cdot \sigma_{\hat{A_{Q}}}^{2} \end{array}\right\}.$$

Inserting the variances, (79) and (80), and the covariance, (85), leads to

(100) $$\mu_{\hat{\varphi }}\approx \varphi +\frac{1}{2}\left\{\begin{array}{c} \left[\frac{1}{A^{2}}\cdot e^{\sigma_{\theta }^{2}}\cdot \sin\left(2\varphi \right)\right]\cdot \left[\frac{A^{2}}{M}\left(1-e^{-\sigma_{\theta }^{2}}\right)+\frac{A^{2}}{2M}\cos\left(2\varphi \right)\left(e^{-2\sigma_{\theta }^{2}}-e^{-\sigma_{\theta }^{2}}\right)\right]+\\ 2\cdot \left[-\frac{1}{A^{2}}\cdot e^{\sigma_{\theta }^{2}}\cdot \cos\left(2\varphi \right)\right]\cdot \left[\frac{A^{2}}{2M}\sin\left(2\varphi \right)\left(e^{-\sigma_{\theta }^{2}}-e^{-2\sigma_{\theta }^{2}}\right)\right]+\\ \left[-\frac{1}{A^{2}}\cdot e^{\sigma_{\theta }^{2}}\cdot \sin\left(2\varphi \right)\right]\cdot \left[\frac{A^{2}}{M}\left(1-e^{-\sigma_{\theta }^{2}}\right)-\frac{A^{2}}{2M}\cos\left(2\varphi \right)\left(e^{-2\sigma_{\theta }^{2}}-e^{-\sigma_{\theta }^{2}}\right)\right] \end{array}\right\}$$

We will now proceed to simplify this rather large mathematical expression. This will be conducted in several simple stages. The first one is to see that, in the first and third lines in the curly brackets, the first term inside the second square brackets cancels out due to the leading minus sign in the third line. We thus obtain

(101) $$\mu_{\hat{\varphi }}\approx \varphi +\frac{1}{2}\left\{\begin{array}{c} \left[\frac{1}{A^{2}}\cdot e^{\sigma_{\theta }^{2}}\cdot \sin\left(2\varphi \right)\right]\cdot \left[\frac{A^{2}}{2M}\cos\left(2\varphi \right)\left(e^{-2\sigma_{\theta }^{2}}-e^{-\sigma_{\theta }^{2}}\right)\right]+\\ 2\cdot \left[-\frac{1}{A^{2}}\cdot e^{\sigma_{\theta }^{2}}\cdot \cos\left(2\varphi \right)\right]\cdot \left[\frac{A^{2}}{2M}\sin\left(2\varphi \right)\left(e^{-\sigma_{\theta }^{2}}-e^{-2\sigma_{\theta }^{2}}\right)\right]+\\ \left[-\frac{1}{A^{2}}\cdot e^{\sigma_{\theta }^{2}}\cdot \sin\left(2\varphi \right)\right]\cdot \left[-\frac{A^{2}}{2M}\cos\left(2\varphi \right)\left(e^{-2\sigma_{\theta }^{2}}-e^{-\sigma_{\theta }^{2}}\right)\right] \end{array}\right\}.$$

We now can observe that the first and third lines in the curly brackets have the same overall sign (the third line has two minus signs that cancel each other). We can thus add the first and third lines together, leading to

(102) $$\mu_{\hat{\varphi }}\approx \varphi +\frac{1}{2}\left\{\begin{array}{c} 2\cdot \left[\frac{1}{A^{2}}\cdot e^{\sigma_{\theta }^{2}}\cdot \sin\left(2\varphi \right)\right]\cdot \left[\frac{A^{2}}{2M}\cos\left(2\varphi \right)\left(e^{-2\sigma_{\theta }^{2}}-e^{-\sigma_{\theta }^{2}}\right)\right]+\\ 2\cdot \left[-\frac{1}{A^{2}}\cdot e^{\sigma_{\theta }^{2}}\cdot \cos\left(2\varphi \right)\right]\cdot \left[\frac{A^{2}}{2M}\sin\left(2\varphi \right)\left(e^{-\sigma_{\theta }^{2}}-e^{-2\sigma_{\theta }^{2}}\right)\right] \end{array}\right\}.$$

We can now see that the two lines are symmetric if we note the minus sign in the second one and the fact that we can swap the order of the sin(2φ) and cos(2φ) terms. Their sum is thus null, and we reach

(103) $$\mu_{\hat{\varphi }}\approx \varphi ,$$

showing that the expected value of the estimator, $\mu_{\hat{\varphi }}$, is equal to the real value that we are estimating, that is, φ. The conclusion is thus that this estimator is unbiased in the presence of phase noise and/or jitter:

(104) $$e_{\hat{\varphi }}=\mu_{\hat{\varphi }}-\varphi \approx 0.$$

Along with the derivation carried out here, we used a Taylor series approximation where only the first term was kept. In order to validate the adequacy of this step, it is necessary to carry out some numerical simulations. This will be conducted in the next section using a Monte Carlo type of procedure.

## 5. Monte Carlo Validation

In order to validate the correctness of the derivations presented and to justify their domain of applicability, several numerical simulations have been carried out, through which the initial phase of a sinewave has been estimated. These simulations involved creating a set of equally spaced data points that follow a given mathematical model: in the present case, the model of a sinusoidal signal. In this model, we included the non-ideal effects we are interested in. For the current purpose, these include the presence of phase noise and jitter in the sampling instant. To this effect, we sampled from a random variable with a specific statistical distribution. Here, we focus on a normal distribution with different values of standard deviation. The use of this type of statistical distribution is justified considering that most sources of phase noise are of thermal origin.

Those values of phase noise were then added to the sampling instants multiplied by the angular frequency, and the value of the sinewave at the resulting time instants was obtained. At this point, one could add the effect of other non-ideal phenomena that might affect the signal, like additive noise or quantization error, but that was not the case in the current study; the only mathematically modeled non-ideal effect was the random phase noise following a normal distribution.

With those corrupted data points from the created sinewave in hand, we employed the least squares three-parameter sine fitting procedure we are studying here and obtained an estimate of its parameters, namely, the amplitude, offset, and initial phase. In the present work, we focus solely on the estimation of the initial phase.

By varying the different parameters of the model, we can see how they affect the estimates. In the current work, we are studying the bias that might be induced in the initial phase estimation of the sinewave. We can then compare those results with the expected ones that we derived analytically.

**Figure 1:**
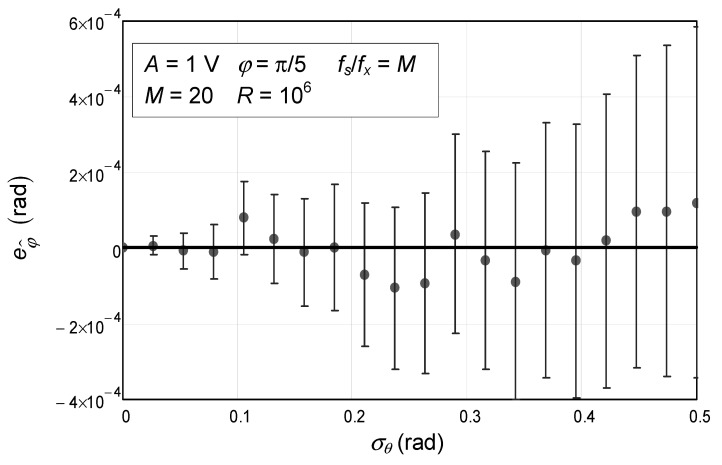
Numerical simulation of the error of the estimated initial phase of a sinewave with 1 V amplitude and an initial phase of π/5 as a function of the phase noise standard deviation. The circles represent the values obtained via Monte Carlo analysis. The confidence intervals for a confidence level of 99.9% are represented by the vertical bars and were obtained with 10^6^ repetitions (*R*). The solid line represents the theoretical value, which is null. This situation is for a case where 20 samples are acquired (*M*).

Focusing on the case in point, we studied the influence of the phase noise standard deviation, $\sigma_{\theta }$, on the systematic error (bias) of the initial phase estimation of a sinusoid, $e_{\hat{\varphi }}$. The results are plotted in Figure 1. The solid circles represent the estimated average value of the initial phase error. In this specific instance, chosen as illustrative, we simulated a sinusoidal voltage signal with 1 V amplitude and an initial phase of π/5 rad. The number of samples acquired was 20 (*M*) and they were acquired at a rate where they covered exactly one signal period by making the ratio between sampling frequency ($f_{s}$) and signal frequency ($f_{x}$) exactly equal to the number of samples:

(105) $$\frac{f_{s}}{f_{x}}=M.$$

The vertical error bars were computed for 10^6^ repetitions (*R*). The higher the number of repetitions carried out, the smaller the error bars become. Since the absolute value of the quantity being estimated (initial phase error) is null, there is not an obvious value that would allow one to say that the error bars are small enough to justify the claim that the estimation is unbiased. Alternatively, we can use the value of the phase noise standard deviation. Since a range of values up to 0.5 rad was used and given that the error bars have a length that increases with phase noise standard deviation and are, in the worst case, smaller than 0.001 rad (where $\sigma_{\theta }$ is equal to 0.5 rad), we might claim that the error bars’ lengths are 500 times smaller than the phase noise standard deviation. Using more repetitions would increase the duration of the simulation without arguably leading to different conclusions.

The theoretical expected values were drawn in the same chart using a thick solid line. In this case, all the points have a null value. As we can see in Figure 1, the error bars are all close to this line, which shows that the result derived agrees with the Monte Carlo simulation.

**Figure 2:**
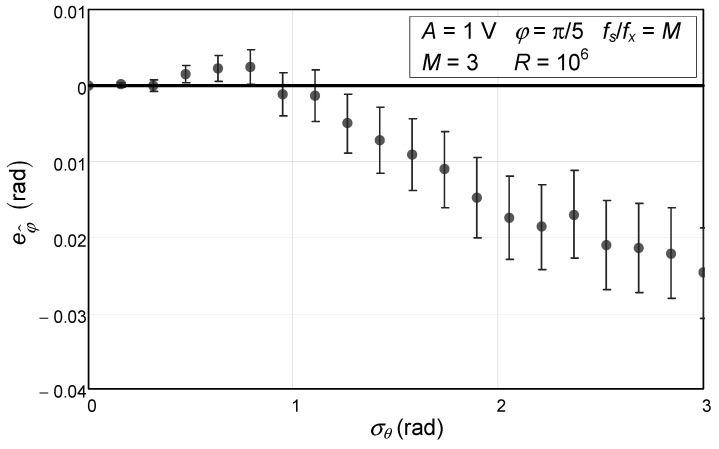
Numerical simulation of the error of the estimated initial phase of a sinewave with 1 V amplitude and an initial phase of π/5 as a function of the phase noise standard deviation. The circles represent the values obtained via Monte Carlo analysis. The confidence intervals for a confidence level of 99.9% are represented by the vertical bars and were obtained with 10^6^ repetitions (*R*). The solid line represents the theoretical value, which is null. This situation is for a case where three samples are acquired (*M*).

As an extreme case, we repeated the same experiment with an extremely low number of samples: just three, which is the minimum possible since we are estimating three parameters (amplitude, offset, and initial phase). We also simulated an amount of phase noise that goes up to 3 rad, which is almost half the sinewave period. This situation, with such a low number of samples and so much phase noise, is not usually encountered in practice and is only intended to show the limits of our model. In this case, the first-order Taylor series approximation made in (18) is obviously not enough, as seen in Figure 2.

Where the phase noise standard deviation is low, the error bars are around 0, but for values higher than 0.3 rad, they are not.

**Figure 3:**
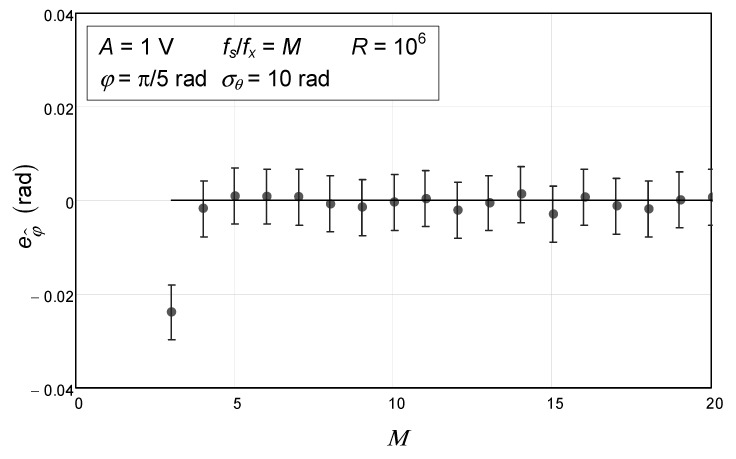
Numerical simulation of the error of the estimated initial phase of a sinewave with 1 V amplitude and an initial phase of π/5 as a function of the number of samples for a phase noise standard deviation of 10 rad. The circles represent the values obtained via Monte Carlo analysis. The confidence intervals for a confidence level of 99.9% are represented by the vertical bars and were obtained with 10^6^ repetitions (*R*). The solid line represents the theoretical value, which is null.

To further study this behavior and its dependence on the number of samples, we carried out a third Monte Carlo simulation where the number of samples varied from three to twenty in the specific case of a very large phase noise standard deviation of up to 10 rad, which is much higher than the usual value encountered in practical situations. The results are depicted in Figure 3. As can be seen, only the first error bar, corresponding to three samples, is outside the theoretical value of 0.

**Figure 4:**
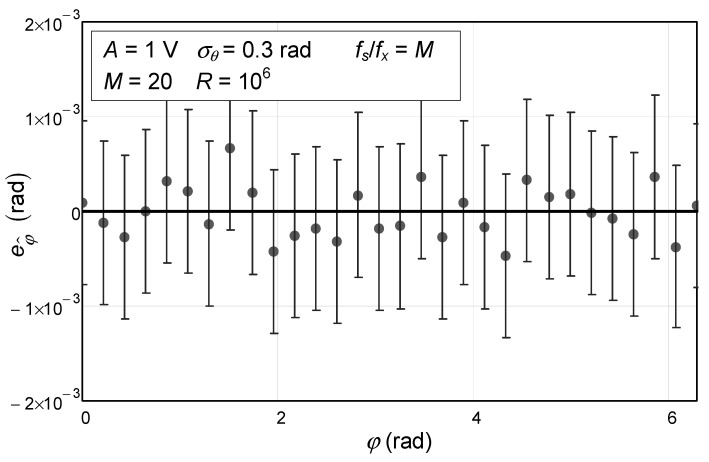
Numerical simulation of the estimated initial phase as a function of the phase noise standard deviation. The circles represent the values obtained via Monte Carlo analysis. The confidence intervals for a confidence level of 99.9% are represented by the vertical bars. The solid line represents the theoretical value, which is null.

Finally, a fourth numerical simulation was carried out where the phase noise standard deviation varied, and the number of samples used was 20 (the other parameters remained the same). The results can be seen in Figure 4.

In this case, we can see that, once again, the error bars of the data points are all close to the theoretical value of 0, as expected, which once again validates the final analytical results that were given in expression (104).

## 6. Conclusions

In this work, we studied how the bias of the least squares estimation of the initial phase of a sinewave depends on the amount of phase noise and jitter that affects the signal. The detailed analytical derivations presented have shown that the bias is null in these particular circumstances. In previous studies, it has also been shown, for example, that the presence of additive noise that the initial phase estimation is not biased [21]. In the case of the amplitude estimation of a sinusoid, however, it has been shown that it is biased in the presence of additive noise [10] and also in the presence of sampling jitter [14].

This type of knowledge is important in order to statistically characterize the measurements made regarding the initial phase of a sinusoid and, together with information about its precision, can be used to define a confidence interval for the estimated sinewave initial phase. We were thus able to show that phase noise and jitter do not bias the estimation of the initial phase of a sinewave.

Recall from Section 2 that three assumptions were made that if not met could render the conclusions of this work invalid; i.e., if those assumptions are not valid, the initial phase estimation may be biased. Those assumptions were: (i) that the data points are homoscedastic, that is, a constant value of phase noise standard deviation is maintained; (ii) that the values of phase noise of different samples are uncorrelated; and (iii) that the data points cover at least an entire period of the sinewave.

Even a null result like this is relevant in that it allows for this type of signal processing technique and estimation to be used confidently and in full knowledge of what does and does not affect it. Note, however, that the derivations made were approximate in as much as they employed only the first term of the Taylor series expansion in (18). For this reason, a numerical simulation was carried out using a Monte Carlo procedure. The results of this simulation allow us to conclude with a high degree of confidence that the estimator is indeed unbiased in typical situations.

This study has also shown that for extremely high phase noise standard deviation values and very small numbers of samples, a bias in the estimation of the initial phase does occur, as demonstrated by the numerical simulations carried out. In essence, what these results tell us is that an approximation using only the first term of the Taylor series expansion is insufficient for this particular case. This is important when statistically characterizing the estimations made and adding information about the expected bias of the results. It also serves as a warning of the dangers of using an unusually small number of samples. However, this work did not set a limit for the error, as it was considered to occur in situations that do not often occur in practice.

## Data Availability

The data presented in this study are available on request from the corresponding author.
